# Treatment of Sciatica

**Published:** 1856-02

**Authors:** Peyton Blakiston


*Treatment of Sciatica.* By Peyton Blakiston. F.R.S.

Dr. Blakiston has pursued the following treatment for twenty years with considerable success. He first saw it adopted in Pars in 1833: A blister, about the size of a crown-piece, is placed over the chief seat of pain, which is usually the flattened part of the buttock. After it has risen well, and the cuticle has been throughly removed, the raw surface is sprinkled with a powder, consisting of one grain of acetate of morphia on an average, and a little white sugar. This dressing is repeated for six successive days, the surface of the blister being kept in a raw state, if requisite, by cantharides or savine cerate, or else by Albuspeyeres' plaster. This suffices for a very mild case; but in severe cases of old standing, the pain will now be found to have left its original seat, and to have seized on the knee of the affected side. The same treatment is then applied to the ham; and after six dressings, the pain will have generally disappeared, and the patient will rapidly recover. By this mode of treatment, 83 cases of uncomplicated sciatica have been cured, without a failure having come to the knowledge of the writer. This number might have been greatly augmented had it included the results arrived at by such of his friends and former pupils as had employed it at his suggestion, and which have been no less successful than those which occurred in his practice; but he is desirous of recording such only as have come under his own immediate notice, and for the accuracy of which he consequently can hold himself responsible. In the great majority of these cases no other drug was administered; but in a few, some laxative medicine or injection was given to remove constipation. In two or three cases, there was a tendency to double sciatica, and then the pain passed from the sciatic region first treated to that of the opposite side, and from thence down to the knee of this last side, but never attacked the knee of the side first affected. It is right to mention, that in hospital practice three cases were placed under the writer's care, which he considered more than doubtful, and they were therefore treated under protest, so to speak. They all turned out cases of hip disease, and therefore they are not included in those above enumerated. The difference in the sensations felt by the patients on the first application of the morphia was remarkable; and without any attempt to generalise, it may be stated that a close connexion was observed between the sensations felt and the previous state of health. Thus the effect produced on three persons in robust health—a blacksmith, a gamekeeper, and a lady—was most intense; an extraordinary thrilling was felt over the whole body, particularly at the extremities, with great nausea, and a tendency to faint. The lady vomited incessantly for twelve hours, so that it was found advisable to reduce the quantity of morphia in the powders to half a grain. On the other hand, a gentleman, who had been much reduced by overwork and by long suffering, felt no effect whatever from the application of the powders, and yet he recovered in an equally short time with the others. A lady, also, who had been taking considerable doses of opium, hardly felt the application of morphia until it was increased to two grains; but this case has been excluded, because, although the sciatica was removed by the treatment, there remained an incurable disease, which eventually destroyed her. One lady, aged 26, in whom the disease was not of long standing, obstinately refused to have a second powder applied; but happily the one application sufficed to effect a cure. In six cases the disease recurred after an interval of from five to eighteen months; and in two of these it recurred twice; but each attack was less severe than the one which preceded it and yielded readily to the same treatment. It is possible, however, that relapses might have more frequently taken place without having come under the notice of the writer; but he thinks this cannot have happened very often. Some other forms of neuralgia were also benefited by this mode of treatment. Thus a very distressing case of neuralgia of the scalp yielded at once; and shooting pains, which frequently accompany cancer of the stomach, were sometimes much relieved by it.—*Med. Times and Gaz.*, *in Dublin Med. Press.*

---

*Illustrations of the Influence of Pregnancy in controlling or retarding the Development of certain Diseases.** By W. F. MONTGOMERY, M.D., Professor of Midwifery to the King and Queen's College of Physicians.

On a former occasion, when treating of the condition of pregnancy, I took occasion to remark that it appeared from experience, that women who bear children generally enjoy more even health and are less disposed to disease that those who lead a life of celibacy, or who, having married, remained unfruitful; so that Gardien seems to express no more than the truth when he says, "Des qu'une femme est grosse les probabilites de sa vie augmentent;" and this is what we ought, *a priori*, to expect, because, childbearing being the ordinance of an all-wise Providence, we should anticipate that the fulfilment of the duty thus ordained would conduce to the welfare of those on whom it has been devolved.

It seems in conformity with such a view to believe, what indeed, I think, experience has taught us, that pregnancy acts in a great degree as a protection against the reception of disease, and perhaps on the common principle, that during the continuance of one very active operation in the system, it is thereby rendered less liable to be invaded or acted on by another; thus it has been observed, that during epidemics of contagious diseases of different kinds, a much smaller proportion of pregnant women have been attacked than of others: but when attacked, they suffer severely; thus, when the cholera visited this country, the proportion of pregnant women who took the disease was very small, but all who caught it died, I believe, without almost a single exception. Gardien's experience led him to a similar conclusion, he says: "Les femmes enceintes sont moins exposees agagner les maladies contagieuses; mais lorsqu' elles ensont atteintes, elles succombent plus promptement." I think also I have seen sufficient to satisfy me that pregnancy does, at least occasionally, exercise another kind of influence over disease in the system, namely, of preventing its development during that state, although the infection may have been caught; as is proved by the disease showing itself immediately after delivery, as in the following cases:—

Mrs. W., when in the ninth month of pregnancy, was much about her brother, who was dangerously ill of malignant scarlatina; she seemed to have escaped the danger completely, but the day after her delivery she was covered with the disease, of which she died in a few days; between the time of her exposure to the infection and her delivery, there had intervened three weeks, during which she appeared to be quite well.

When Mrs. F. was in the eighth month of pregnancy, her husband had typhus fever, in which she assiduously attended

* Read before the Association of the College of Physicians of Ireland.

him; after his recovery, she went to her father's house, some fifty miles from town, where she was delivered in due time, and immediately afterwards was seized with typhus fever, of which she died, in eight days; between five and six weeks had elapsed between Mr. F.'s illness and her labor, and during that interval, she seemed in perfect health.

In the month of November, 1854, I attended a young lady in her first confinement; previous to which she had both the lower extremities much enlarged by anasarca, but she appeared, in other respects, quite well, with one exception, which was that she had such *soreness* of the abdomen, she found a difficulty in laying on either side; and when I passed my hand over the abdomen, she complained that the pressure hurt her everywhere. On the 12th she was confined, after a favorable labor, but the abdominal tenderness remained, and there was a peculiar doughy feel of the whole abdomen; next day, this was equally felt, but with little or no pain or fever, and a perfectly quiet pulse.

On the 14th, I found the insteps of both feet, but particularly the left, covered with well-developed erysipelas; her mother, who seemed very anxious about her, was present when I examined the feet, and on our reaching the drawing room said, "Doctor, isn't that very like erysipelas?" I said, "Yes, certainly, there was no doubt about it." "Dear me, sir, do you think she could have taken it from her husband?" She then, for the first time, informed me, that some weeks before leaving home, to come to town for her confinement, her busband had a severe attack of erysipelas, during which she had assiduously nurse-tended him. Immediately on the appearance of the erysipelas on the feet, the abdominal symptoms began to decline, and, after two or three days, ceased to exist. I cannot but believe that this lady caught the infection from her husband during her close attendance on him, that it remained in abeyance until gestation was over, and was then developed. She recovered well.

It is, I believe, a matter of common observation, that when women who have been laboring under certain forms of disease happen to conceive, the morbid affection previously existing is oftentimes either greatly mitigated, checked, or even altogether suspended for a time, as has been frequently observed in persons affected with phthisis; though I must add that the influence of pregnancy in cases of phthisis is a question on which a variety of discordant opinions has been given by high authorities. Andral's conclusion, from his latest observations, is "that in the great majority of cases the symptoms of phthisis are suspended, or at least remain stationary during the course of pregnancy."

Louis says he is not "in a condition to determine whether pregnancy is, or is not capable of retarding the progress of phthisis," but he suggests that the fact might be, that several of the symptoms become somewhat more obscure during pregnancy, without any check being in reality given to the advance of the disease. My own experience would lead me to the conclusion, that if a woman predisposed to phthisis, but in whom the disease has not actually become developed, prove pregnant, she is likely to be benefitted thereby; and I think I have seen life thus prolonged, for years, in several instances; but, on the other hand, if pregnancy takes place in a woman already actually in consumption, or if this disease supervene on pregnancy, the fatal issue is as likely to be accelerated as postponed, or, perhaps, even more so.—*Dublin Quarterly Jour. Med. Sci.*

---

British Mortality in England and the Crimea.—If all the deaths of British soldiers in the Crimea during the last three months were added to the deaths in England, the sum would be less by some twenty thousand than the deaths registered in England during the three summer months of 1854. In fact, it results from the Report of the Registrar General, for the Quarter ending September, 1855, that the summer months of the present year have been unusually favorable as regards health, notwithstanding the undoubted disadvantage under which the poor have suffered in respect to the price of provisions. The causes assigned for the improvement in the public health, by the District Registrars, are various, but they are quite in unison in respect to the inestimable advantages accruing from sanitary improvements. The deaths in the summer quarters of the five years 1851–55 were as follows:—

| 1851. | 1852. | 1853. | 1854. | 1855. |
|---|---|---|---|---|
| 91,499 | 100,382 | 92,201 | 113,939 | 87,934 |

It is a curious circumstance, in reference to the means of livelihood of the people, that while in the interval of two years the price of wheat has risen 48 per cent., and that of beef 15 per cent., there has been a fall of 37 per cent. in the price of potatoes, an article of large consumption among all classes, and which has operated as a set-off against the high price of other articles; thus, perhaps, partly accounting for the fact that the public health is better than in the summer of 1853. In reference to the Meteorology of the quarter, Mr Glaisher reports that the departures from the average values of the temperature have in no cases been large during the quarter, and that other subjects of investigation have been about their average. Such being the facts of the case, it remains only that, eschewing the insignificant few who, for peculiar reasons, are *sans* olfactories, we steadily pursue the work of sanitary reform, having confidence in the aphorism, "*Aide-toi, et le Ciel t'aidera.*"—*London Medical Times.*

---

DURATION OF LIFE.

| | Yrs. | Mos. |
|---|---|---|
| The average age of all alive above 15, in America, is | 33 | 6 |
| The average age of all alive above 15, in England and Wales, is . . . . . . . . . . . . . . . . | 37 | 5 |
| The average age of all above 20 years, in America, is | 37 | 7 |
| In the whole of England, the average of all above 20 years, is . . . . . . . . . . . . . . . . | 41 | 1 |

"These statements are important, and, coming from a man so eminent for the ability and knowledge he has displayed on this subject, deserve serious consideration.

"It is the prevailing opinion among us, that no people in the world are more healthy than ourselves; but if the above statements are true, this opinion is erroneous.

"In one of the Massachusetts Reports, is a compilation showing the mean duration of life in several places in Europe; also, in Massachusetts. From this it appears, that while a child has a chance of living 45 years in Surrey (one of the healthiest districts in England) it has a chance of living only 25 years in Liverpool, and 28.15 years in Massachusetts—showing a difference between the two first of 19.6 years; in other words, life is but five-ninths as long in Liverpool as in Surrey. Yet before the facts developed by the registration system were known, it was asserted by one of the most accurate writers in England, that the great increase in the town of Liverpool was owing *to the salubrity of the air*, and the progressive improvement in trade, commerce, and steam navigation.

"If the above statement, as to the mean duration of life in Massachusetts, be correct (which is doubtful) it is as unhealthy as Liverpool and the most unhealthy districts of England. Facts as interesting and important may yet be developed in this State, in relation to our own towns and villages. We are becoming largely a manufacturing people, and take pains to ascertain the exact cost of every article made, in all its different parts, and its cost of transportation; yet we know nothing of the cost of life involved in its production."

